# Sparsification of long range force networks for molecular dynamics simulations

**DOI:** 10.1371/journal.pone.0213262

**Published:** 2019-04-12

**Authors:** Peter Woerner, Aditya G. Nair, Kunihiko Taira, William S. Oates

**Affiliations:** 1 Department of Mechanical Engineering, Florida A&M-Florida State University College of Engineering, Tallahassee, FL, United States of America; 2 Department of Mechanical Engineering, University of Washington, Seattle, WA, United States of America; 3 Department of Mechanical and Aerospace Engineering, University of California Los Angeles, Los Angeles, CA, United States of America; Danish Cancer Society Research Center, DENMARK

## Abstract

Atomic interactions in solid materials are described using network theory. The tools of network theory focus on understanding the properties of a system based upon the underlying interactions which govern their dynamics. While the full atomistic network is dense, we apply a spectral sparsification technique to construct a sparse interaction network model that reduces the computational complexity while preserving macroscopic conservation properties. This sparse network is compared to a reduced network created using a cut-off radius (threshold method) that is commonly used to speed-up computations while approximating interatomic forces. The approximations used to estimate the total forces on each atom are quantified to assess how local interatomic force errors propagate errors at the global or continuum scale by comparing spectral sparsification to thresholding. In particular, we quantify the performance of the spectral sparsification algorithm for the short-range Lennard-Jones potential and the long-range Coulomb potential. Spectral sparsification of the Lennard–Jones potential yields comparable results to thresholding while spectral sparsification yields improvements when considering a long-range Coulomb potential. The present network-theoretic formulation is implemented on two sample problems: relaxation of atoms near a surface and a tensile test of a solid with a circular hole.

## Introduction

Molecular dynamics (MD) is a common simulation tool for modeling materials at the nano and micron length scales [1, 2]. In the framework of MD simulations, atoms are treated as point masses that are accelerated by an imbalance of interatomic forces from neighboring atoms. To track the atomic trajectories, numerical integration of Newton’s equations of motion is performed which often requires significant computer memory and lengthy computations to account for each interatomic interaction across a large number of time-step integrations. A variety of atomistic scale insights about complex material behavior can be determined including, mechanical [1, 3, 4], chemical [5, 6] and thermal [7–9] properties and in limiting cases, macroscopic constitutive relations.

Many simulations frequently require millions of atoms, making the computations expensive [10]. Moreover, a large number of time steps are required to acquire meaningful thermodynamic statistics [1, 2]. Additionally, accurate dynamic trajectories require time steps on the order of the frequency of atomic vibrations which is typically on the order of femtoseconds. Therefore, simulations on the nanosecond timescale require millions of time steps over millions of degrees of freedom on a three-dimensional grid [1].

Specialized research focused on improving computational speed in MD simulations has focused on parallel implementation [1]. This has led to the creation of both novel algorithms and specialized hardware [1, 11–14]. However, long range forces remain difficult to compute and can suffer accuracy limitations. Typically, interactions above a cutoff distance are neglected (i.e., thresholding). For short range forces, this is often sufficient. However, the effects due to thresholding become uncertain when using long-range forces such as those produced from electrostatic and magnetostatic problems. Broadly speaking, the approaches currently used for dealing with these long range forces can be classified as one of the following: use a cutoff distance despite the potential issues, transform the Coulomb potential into a short range potential, e.g., Debye potential and Wolf summation, or mathematically exploit periodic boundary conditions to generate a mathematical expression long range effects [15–18]. Thresholding is the most computationally efficient of the current methods, but generates large error. The transformation strategy suffers from the same drawbacks as the thresholding strategy; however, it has been shown to be adequate in some applications [18]. Methods for solving the full solution by exploiting periodic boundary conditions are accurate, but computationally expensive [14, 19, 20].

Research focusing on sparsity has recently gained traction in computational science [21–23]. In machine learning, the use of the *l*_1_ norm has been leveraged to promote sparsity in the resulting models without over-fitting [24, 25]. In handling big data, autoencoders and other sparse techniques are used to create sparse representations of data to reduce memory requirements [23, 26]. In network theory, sparsification is used to create computationally tractable representations of complex systems [27]. The use of sparsity to model continuum dynamics has recently emerged to describe nonlinear flow phenomena but there has been limited research in the field of atomistic scale computational materials science [28].

In this paper, we examine the kinematics and dynamics of atomistic systems using network theory. Specifically, we utilize network-based techniques which create computationally tractable representations of the atomic system without sacrificing accuracy. Network theory has long history of describing complex systems such as social interactions, electric grids, and airplane scheduling [29]. Although there have been applications in engineering systems such as chemical networks [30], fluid dynamics [28, 31–34] and granular matter [35, 36], minimal research has focused on applications in molecular dynamics and solid mechanics.

A network consists of nodes or vertices and their pairwise connections which are called edges [29].

Sparse representations of networks are desirable because they distill the network to its core features.

We describe and implement spectral sparsification, a technique for creating sparse representations of networks, on atomisitic networks. This method generates a sparse network while while maintaining spectral similarity with the original network [27, 37]. The results to be presented here demonstrate that spectral sparsification is a viable candidate to enhance computational efficiency of MD calculations while maintaining minimum errors.

Spectral sparsification is applied and implemented to atomic networks using the Lennard–Jones and Coulomb potentials. Spectral sparsification conserves global network properties by reorganizing interatomic force distributions in order to achieve a sparse representation of the interatomic forces. In what follows, we first introduce the essential relations used in molecular dynamics calculations followed by definitions and concepts used in network theory that are relevant to molecular dynamic modeling. We then introduce a formulation of atomic networks and describe the spectral sparsification algorithm. Finally, we present and quantify the error in kinematic and dynamic examples.

## Molecular dynamics

Molecular dynamics calculates the motion of atomic nuclei via Newton’s law

$$m\ddot{\text{x}}=\sum F,$$ (1)

where **F** are the interatomic forces within the solid, *m* is the mass and $\ddot{x}$ is the acceleration [1, 2]. The summation and calculation of **F** comprises the majority of the computational resources in MD simulations. This is the one of the key aspects that motivates the use of a sparse force interaction network in this work.

The forces on the atoms are given by

F=-∇U, (2)

where *U* is the potential energy and $\nabla =\frac{\partial }{\partial r_{\alpha \beta }}$ is the gradient with respect to the relative atomic distance between two atoms. This distance between two atoms *α* and *β* is defined by *r*_*αβ*_ = |***r***_*α*_ − ***r***_*β*_| [2].

The simplest potentials are functions where the only dependent variable is the distance between two atoms, i.e. the pair potential

$$U_{\alpha \beta }=U\left(r_{\alpha \beta }\right).$$ (3)

The vector **r**_*α*_ denotes the absolute position of atom *α*. The potential energy is defined by *U*_*αβ*_. Summing all pairwise potentials yields the total energy.

In the current paper, we consider the Lennard-Jones and Coulomb potentials. The Lennard–Jones potential is given by

$$\begin{array}{c} U_{\alpha \beta }^{LJ}=\epsilon_{0}[{\left(\frac{\sigma }{r_{\alpha \beta }}\right)}^{12}-{\left(\frac{\sigma }{r_{\alpha \beta }}\right)}^{6}], \end{array}$$ (4)

where *ϵ*_0_ defines the depth of the energy well and *σ* defines the zero potential distance.

The Coulomb potential is given by

$$\begin{array}{c} U_{\alpha \beta }^{C}=k\frac{q_{\alpha }q_{\beta }}{r_{\alpha \beta }}, \end{array}$$ (5)

which describes interactions between two point charges where *k* is Coulomb’s constant given by, in S.I. units, *k* ≈ 8.99 × 10^9^Nm^2^/C^2^, and *q*_*α*_ and *q*_*β*_ are the charges of the two atoms [38].

In solid mechanics, stress, potential energy, and kinetic energy are frequently quantities of interest. This paper uses the standard expression for the virial Cauchy stress

$$\begin{array}{c} \sigma \left(x,t\right)=\frac{1}{N_{t}V_{\Omega }}\sum_{t=0}^{N_{t}}(-\sum_{\alpha \in \Omega }m_{\alpha }v_{\alpha }⊗v_{\alpha }+\frac{1}{2}\sum_{\alpha ,\beta \in \Omega }r_{\alpha \beta }⊗F_{\alpha \beta }), \end{array}$$ (6)

where ⊗ denotes a tensor product over the velocity of atom *α* given by ***v***_*α*_. The atomic mass is *m*, the number of time steps chosen for time averaging the stress is *N*_*t*_, and the region which is being considered over the volume *V*_Ω_ is denoted by Ω. The average stress over the volume is defined at the center of mass point **x**. This average is based on a spatial average over the velocity of the *α* atoms within the volume. Similarly, the last summation is over the forces **F**_*αβ*_ for all pairs of atoms *α* and *β* in the domain Ω.

The kinetic energy of the system is calculated by

$$E_{k}=\frac{1}{2}\sum_{\alpha }m_{\alpha }v_{\alpha }\cdot v_{\alpha }$$ (7)

and the potential energy is given by summing up the pairwise interactions

$$U=\frac{1}{2}\sum_{\alpha ,\beta }U_{\alpha \beta }.$$ (8)

The summation *α*, *β* is again taken over all pairs atoms in the system. The last three relations will be used in addition to interatomic forces to quantify the performance of spectral sparsification versus thresholding algorithms. Rather than focusing on the entire stress tensor, we will instead look at the first and second invariants of the stress tensor [39]. The first invariant is given by the trace of the stress tensor

I=tr(σ) (9)

and the second invariant is given by

$$II=\frac{1}{2}[{\left(\text{tr}(\sigma )\right)}^{2}-\text{tr}\left(\sigma^{2}\right)].$$ (10)

Physically, the first invariant is the hydrostatic pressure felt by the material while the second invariant is related to the shear applied to the material which is often used to simulate plastic deformation in solids [39].

Errors are computed by comparing the sparse representations to the corresponding complete graph that contains all long range interactions.

## Network theory

We briefly describe the fundamental concepts from network theory followed by its application to MD simulations and the spectral sparsification algorithm.

### Formulation

Network theory is used to model the interactions between objects [29]. Formally, a graph *G* is an ordered set *G* = (*V*, *E*, *w*(*E*)) consisting of a nonempty set of the vertices, *V* = {*v*_1_, *v*_2_, …, *v*_*N*_}, and edges, *E*, which describe the connections between pairs of vertices and the weights, *w*(*E*), which assigns a nonnegative weight to each edge. If the weights are restricted to one or zero, the graph is categorized as unweighted. Otherwise, the weights can take any nonnegative value and the graph is categorized as weighted. A graph is considered to be undirected if *w*_*αβ*_ = *w*_*βα*_ for all edges in *G*. Otherwise, the graph is called directed.

A common mathematical representation of networks is the adjacency matrix *A* ∈ ℜ^*N*×*N*^

$$A_{\alpha \beta }=\{\begin{array}{cc} w_{\alpha \beta } & \text{if}\;(\alpha ,\beta )\in E\\ 0 & \text{otherwise}. \end{array}$$ (11)

Relevant to this work is the fact that the adjacency matrix of an undirected graph is symmetric.

The graph Laplacian *L* ∈ ℜ^*N*×*N*^ is given by

$$L_{\alpha \beta }=\{\begin{array}{cc} k_{\alpha } & \text{if}\;\alpha =\beta \\ -w_{\alpha \beta } & \text{if}\;(\alpha ,\beta )\in E\\ 0 & \text{otherwise,} \end{array}$$ (12)

where *k*_*α*_ = ∑_*β*_ *A*_*αβ*_ is the nodal strength of *α*. The graph Laplacian and the adjacency matrix play key roles in graph-theoretic algorithms.

### Atomistic networks

To create an atomic network representation, we take the individual atoms as the vertices and the force magnitude as the scalar edge weights. The magnitude of the force between two atoms separated by ***r***_*αβ*_ determines the edge weight,

$$w_{\alpha \beta }=\left|F_{\alpha \beta }\right|.$$ (13)

In this paper, we quantify the network structure with the nodal strength and its distribution [29]. The nodal strength is particularly important in this application because it describes the total force magnitude of each particle. The strength distribution is graphically represented using a histogram plot which can display changes in structure of the graph.

### Atomistic network sparsification

Sparsification involves creating a sparse representation, *G*_*s*_, that approximates the true network, *G*. In this case, sparsity is described by a reduction in the number of edges while keeping the number of vertices fixed. Network similarity is the concept used to compare the sparse representation with the original graph. Different sparsification methods have been described based on different definitions of similarity [40, 41]. Spectral sparsification [27], which defines similarity based on the eigenspectra, is chosen due to its previous success in studying dynamics across networks [28].

For this paper, we define *G*_*s*_ to be similar to *G*, with approximation order denoted by *ϵ* ∈ [0, 1] such that

$$\begin{array}{c} \left(1-\epsilon \right)v^{T}Lv\leq v^{T}L_{s}v\leq \left(1+\epsilon \right)v^{T}Lv \end{array}$$ (14)

for all $v\in R^{N}$ where *L* is the graph Laplacian, *L*_*s*_ is the sparsified graph Laplacian [27]. The approximation order, *ϵ* governs the potential sparsity of the system. If *ϵ* = 0 the algorithm will not sparsify any edges in the network while at the other extreme where *ϵ* = 1, maximum sparsification is allowed by the algorithm. The following steps describe the spectral sparsification algorithm [27, 28] which achieves spectral similarity with probability greater than one half:

1. Calculate the effective resistance given by the formula
  $${\hat{R}}_{ij}={\left(p_{i}-q_{j}\right)}^{T}L^{+}\left(p_{i}-q_{j}\right),$$ (15)
  where *L*^+^ is the Moore-Penrose pseudoinverse of the Laplacian while *p* and *q* are the vector representation of the nodes. For example, the first node label is represented by *p*_1_ = (1, 0, …, 0) = *q*_1_.
2. Select *n*_*e*_ edges randomly with probability proportional to the edge’s effective resistance, ${\hat{R}}_{ij}$. This probability is, $p_{ij}={\hat{R}}_{ij}/\sum_{ij}{\hat{R}}_{ij}$. This selects edges based on their importance to the network. The parameter *n*_*e*_ is given by *n*_*e*_ = 8*N*log_2_(*N*)/*ϵ*^2^.
3. Add the sampled edges to the sparse graph *G*_*S*_ with weight ${\hat{w}}_{ij}=nw_{ij}/\left(n_{e}p_{ij}\right)$, where *q* is the number of samples and *n* is the multiplicity of selection. Edges which are not selected are removed or, equivalently their weight set to zero.

Because spectral sparsification reduces the number of edges, it provides a novel way to reduce the computations of forces while maintaining a low level of error during MD simulations. In particular, the algorithm will be shown to be favorable for long-range forces and estimation of continuum material properties. We kinematically and dynamically implement and test this algorithm on a two-dimensional domain containing a circular hole. We show that spectral sparsification preserves the total force for long-range potentials. This result is compared to the frequently used thresholding method which fails to preserve the total force. Because of this, we consider spectral sparsification as a simple alternative to other methods, e.g. fast multipole method [20, 42–45] and the Wolf summation method [16] which also preserve the total force magnitude.

### Spatial domain decomposition

It is known that thresholding can be accomplished in O(n) time via spatial decomposition [1] while sparsification does not scale as effectively (see Section A in S1 File). This section describes an algorithm with better scaling properties. In particular, we present an algorithm which combines thresholding and spectral sparsification with superior scaling properties to the original spectral sparsification. We begin by describing a modification to the spatial domain decomposition used in thresholding for spectral sparsification.

Here, we take advantage of the O(n) scaling of the thresholding algorithm by partitioning the domain into subdomains with size slightly larger than the cut-off distance. This allows the algorithm to only look for pairs in the neighboring subdomains. This builds upon previous work that has shown how sparsification can be performed on subgraphs of the original graph [46]. Therefore we can divide the domain spatially into subgraphs which can be individually sparsified and can also facilitate parallelization of the algorithm.

Domain decomposition for spectral sparsification first divides the original graph *G* into subgraphs *G* = ∪*G*_*i*_, then approximates the subgraphs via sparsification $G_{i}\approx {\tilde{G}}_{i}$. The sparsified subgraphs (${\tilde{G}}_{i}$) are recombined to form an approximation of the original graph, i.e. $G\approx \cup {\tilde{G}}_{i}$. From a numerical standpoint, the algorithm partitions the adjacency matrix into submatrices and creates sparse approximations of the submatrices which are reassembled to generate a sparse approximation of the original matrix.

The key steps associated with domain decomposition include:

1. Partition the domain Ω into subdomains {Ω_1_, …, Ω_*m*_}.
2. Sort the atoms into each subdomain.
3. For each subdomain, Ω_*i*_, create a graph of the atomic interactions within the subdomain.
4. Sparsify this subgraph.
5. For each pair of subdomains, (Ω_*i*_, Ω_*j*_), generate the atomisitc graph consisting of the atomic interactions of all atoms in both subdomains.
6. Sparsify the subgraph consisting of all interactions in Ω_*i*_ and Ω_*j*_, then remove all edges which do not contain a node in both subdomains.
7. Reassemble all approximations of the subgraphs together to create the approximation of the original graph.

### Combined algorithm

Our combined algorithm consists of using the positive attributes of both thresholding and sparsification. Sparsification is performed via domain decomposition described in the previous section after thresholding using a prescribed cutoff radius. There is an important tradeoff to be mentioned. As the cutoff radius shrinks, sparsification removes fewer edges and the algorithm gives results similar to thresholding. In the other direction where the cutoff radius increases, the algorithm becomes similar to pure spectral sparsification and approaches pure spectral sparsification in the limit of an infinite cutoff radius.

## Kinematic implementation and results

**Fig 1:**
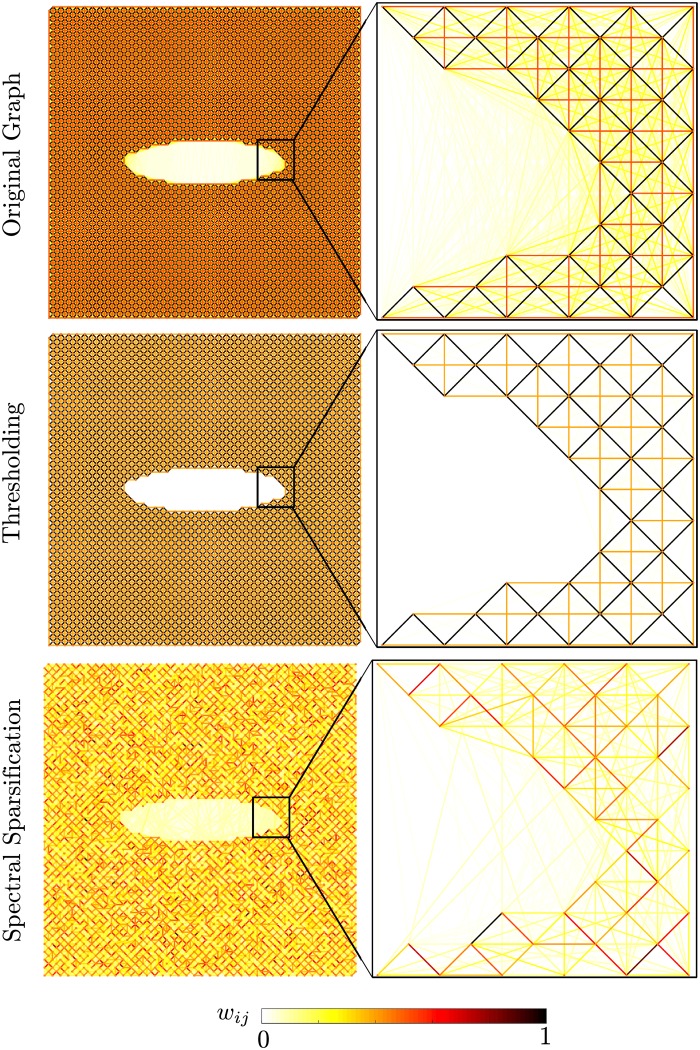
Coulomb force graph with thresholding and with spectral sparsification. The color represents the normalized edge weight.

The present analysis evaluates spectral sparsification by investigating a test problem with the Coulomb potential arranged in a two-dimensional crystalline material with an elliptical void space (see Fig 1). Additional comparisons to a circular hole can be found elsewhere [47]. This test problem was chosen because it is relatively simple and evaluates spectral sparsification for problems with an elliptical hole defect. We compare example graphs by varying the edge densities generated by evaluating the radial cutoff length of thresholding and changing *ϵ* as given in (14) for spectral sparsification.

We define near-equilibrium position as the minimum energy lattice points for a perfect, infinite crystal. Near-equilibrium position, therefore, neglects surface relaxation on the boundary of our finite crystal and the free surface around the hole. The model consists of a square with 30 unit cells where atoms near the center have been removed.

We focus on the effect of thresholding and sparsification by evaluating long range forces from the Coulomb potential in Fig 1. In this figure, we plot the network representing the original graph, the thresholded graph for a cut-off radius *r*_*cut*_ = 10*σ* (*σ* given in (4)), and the spectral sparsified graph with *ϵ* = 1. Fig 1 illustrates the differences in sparsified forces for the long-range Coulomb potential. An important difference in the algorithms is demonstrated here. Spectral sparsification naturally detects and retains important long range forces. This is visualized by noticing that both algorithms cut interactions across the hole. However, thresholding removes all of these interactions while spectral sparsification maintains a finite amount of edges while still creating a sparse representation.

The number of nonzero entities of the adjacency matrix representing interatomic force interactions is illustrated for models exhibiting similar error levels; see Fig B in S1 File. These error measures are defined later in (18). The left image in Fig B in S1 File applies the Coulomb potential with a cutoff radius of 15*σ* while the right image in Fig B in S1 File applies the Coulomb potential which spectrally sparsifies the graph using *ϵ* = 1. Further comparisons are given in Fig C in S1 File where the adjacency matrices of a 200 atom system for varying levels of sparsity is visualized. We see that spectral sparsification retains some of the long range forces and removes some of the medium length forces in comparison to the thresholded adjacency matrix. This highlights one key advantage of spectral sparsification: it retains sufficient long range interactions to capture long range effects while strategically removing edges for computational efficiency.

**Fig 2:**
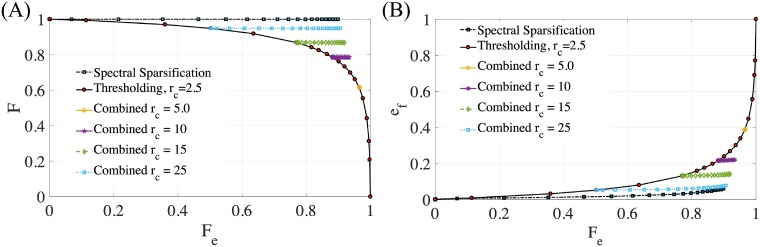
Evaluation of the Coulomb potential on total forces and force errors versus edges removed. (A) Net force vs. fraction of edges removed for thresholded, sparsified, and the combined algorithm. (B) Normalized error vs. edges removed for thresholded, sparsified, and the combined algorithm.

We focus on the error analysis of the kinematic problem by examining the long range Coulomb forces and provide comparisons to the Lennard-Jones potential summarized elsewhere [47]. The number of edges removed is varied by adjusting the cutoff radius in the case of thresholding and varying the value of *ϵ* for spectral sparsification. The net normalized forces versus the fraction of edges removed is plotted in Fig 2A. The net normalized force, $\tilde{F}$, is defined by

$$\tilde{F}=\frac{\sum_{ij}A_{ij}^{s}}{\sum_{ij}A_{ij}},$$ (16)

where $A_{ij}^{s}$ and *A*_*ij*_ are the adjacency matrices of the sparsified and original graphs, respectively. The net normalized force represents the magnitude of all the force interactions in the network. Edge sparsification is quantified by the fraction of remaining edges *F*_*e*_ which is evaluated by

$$F_{e}=1-\frac{N_{e}^{s}}{N_{e}^{o}},$$ (17)

where $N_{e}^{s}$ and $N_{e}^{o}$ are the number of nonzero edges in the sparsified graph and the original graph, respectively.

In addition to the total force analysis, we also show the average normalized error of the nodal strength. This error is equivalent to the error of the total normalized force on each atom which can be calculated from

$$e_{f}=\frac{1}{N∥A_{ij}∥}∥\sum_{i}A_{ij}^{s}-\sum_{i}A_{ij}∥,$$ (18)

where ∥ ⋅ ∥ represents the Euclidean norm and *N* is the number of nodes.

This error describes the change in the magnitude of forces on each atom. In prior analysis, we have shown that both spectral sparsification and thresholding introduce relatively small errors until *F*_*e*_ ∼ 0.98 for the Lennard–Jones potential [47]. For cases *F*_*e*_ > 0.98, it was shown that thresholding performs slightly better until *F*_*e*_ ≥ 0.99. This suggests that thresholding is more accurate than spectral sparsification for short range forces.

In Fig 2, we illustrate the total force and error analysis for the Coulomb potential. It is shown that sparsification outperforms thresholding by cutting and redistributing edges to conserve global forces. We see in Fig 2A that sparsification maintains the global net force, whereas errors using thresholding increase at a faster rate as *F*_*e*_ → 1. Furthermore, in Fig 2B we see that spectral sparsification has lower local error than thresholding for the same fraction of edges removed. This suggests that the use of spectral sparsification is efficient at removing edges for models with long-range forces since lower error tolerances can be achieved with the same number of edges removed relative to thresholding. Additionally, Fig 2 shows that combining thresholding and spectral sparsification allows one to first select a point on the error vs. thresholded distance curve and then remove more edges without significantly changing the net force and the error (i.e. local force). This demonstrates that combining spectral sparsification with thresholding gives the best of both algorithms: better scaling due to thresholding combined with fewer edges to preserve global force accuracy due to sparsification.

We emphasize that spectral sparsification maintains the mean nodal strength distribution while achieving a low level of global errors. In contrast, thresholding discards edges instead of redistributing them and therefore fails to maintain the mean nodal strength. Qualitatively, spectral sparsification keeps the average nodal strength constant while broadening the distribution of the nodal degree. While the distribution of the nodal strength contains randomness, the algorithm maintains a similar Laplacian eigenvalue spectrum and corresponding eigenvectors. Maintaining these two quantities should maintain global dynamic properties. Therefore, from a kinematic perspective, spectral sparsification better approximates the forces compared to thresholding.

**Fig 3:**
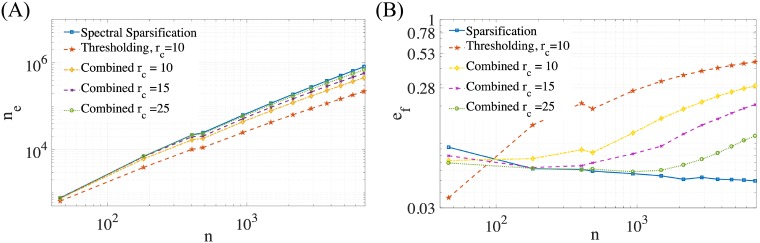
(A) The number of edges as a function of atoms for sparsification, thresholding, and the combined algorithm for multiple cut-off radii (thresholding was done at *r*_*c*_ = 10*σ*). (B) Kinematic estimation of local (force) error as a function of number of atoms in the system.

We also conduct numerical scaling experiments with the Coloumb potential to evaluate the performance of spectral sparsification. These examples are run using the same geometry as our previous kinematic example by varying the number of atoms from 10 to 10^4^. The number of edges and the kinematic force error is compared for spectral sparsification, thresholding, and the combined algorithm. The scaling on the number of edges is tested for several cutoff radii as indicated in the figure. Spectral sparsification used *ϵ* = 1 for all simulations. We note that a cutoff radius *r*_*c*_ = 15*σ* (*σ* = 1 in our model) has been chosen based on previous work which suggest this *r*_*c*_ is a reasonably appropriate cutoff radius for the Coulomb potential [48, 49]. Results are shown in Fig 3. The number of edges appears to scale linearly in Fig 3A, which is better than the O(nlog(n)) scaling proven in [27]. Additionally, we find that the local error for sparsification scales better than for thresholding. We note that the level of error in the combined algorithm is set by the degree of thresholding.

Given the complexity reduction and computational scaling (see Section A and Fig A in S1 File for more details), it is preferred to limit the number of times sparsification is applied during molecular dynamic time integration simulations. We investigate the effect of sparsification using thresholding and spectral methods in the following section to quantify error propagation for a fixed amount of sparsification applied at the initial time of the simulation. We conduct these simulations on a circular hole to simplify the analysis. The sparsification is fixed in all simulations such that we can quantify the amount of error that propagates over multiple time steps of numerical integration.

## Molecular dynamics implementation

For time stepping, we use the traditional Verlet algorithm given by

$$\begin{array}{cc} r^{\left(i+1\right)} & =r^{\left(i\right)}+v^{\left(i\right)}\Delta t+\frac{\Delta t^{2}}{2}a^{\left(i\right)},\\ v^{\left(i+1\right)} & =v^{\left(i\right)}+\frac{\Delta t}{2}(a^{\left(i+1\right)}+a^{\left(i\right)}), \end{array}$$ (19)

where Δ*t* is the time step, ***r***^(*i*)^ is the position of the atoms, ***v***^(*i*)^ is their velocity, and ***a***^(*i*)^ is the acceleration at the *i*th time step. The acceleration is computed from the negative gradient of the potential energy normalized by the atomic mass according to (1) and (2).

Two different types of errors are computed. The first is the atomic trajectory or local error. This is calculated by taking the difference in positions between an exact simulation where neither thresholding nor sparsification is applied and compared to an otherwise identical simulation where an approximation of the force is used through one of the algorithms: spectral sparsification, thresholding, or the combined algorithm. The other error metric considered is global quantities of interest including the pressure, the second stress invariant, the kinetic energy, the potential energy, and the total energy.

Spectral sparsification used in this analysis requires all entries in the adjacency matrix to be positive. Incorporating both negative and positive charges into this formulation may be possible, but is beyond the scope of the current algorithm development [33]. Further research is required to understand how both positive and negative charges may be sparsified. In order to demonstrate the efficacy of spectral sparsification in molecular dynamics, we restrict the dynamic implementation to problems with only positive charges for the present investigation.

Two example problems are considered using a potential which combines the Coulomb and the Lennard-Jones potentials. The Lennard-Jones potential is included for model stability. This resembles the potentials used for modeling ionic solids. The results presented here use Lennard-Jones reduced units, i.e., units where *ϵ*_0_ = 1 and *σ* = 1 with the timescale based on the angular frequency of small oscillations in a Lennard-Jones oscillator given by

$$\omega_{0}=\sqrt{\frac{72\epsilon_{0}}{2^{1/3}m\sigma^{2}}}.$$ (20)

For reference, this equation is derived in Section B in S1 File. The magnitudes of the charges have been scaled such that the repulsive forces are strong enough to be significant while small enough to not cause the material to rupture. Specifically we set $q=0.1\sqrt{\epsilon_{0}\sigma /k}$ where *k* was given earlier in (5) as Coulomb’s constant.

While periodic boundary conditions are ubiquitous in molecular dynamics, this paper primarily deals with defects associated with free boundary conditions. The boundary conditions are chosen so that comparisons are made to the algorithm itself and not necessarily due to issues involving periodic boundary conditions. The first example is relaxation of a solid along free boundaries. The second problem is a tensile test of a solid with a circular hole in the side using half symmetry boundary conditions (a classic problem in elasticity).

**Fig 4:**
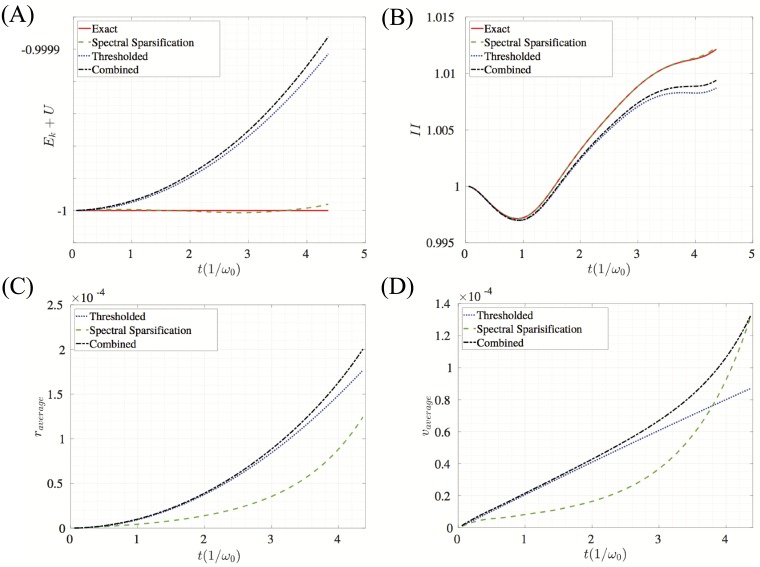
Measures of error and global properties for the sparsified, thresholded, and combined approximations for the surface relaxation problem. Comparisons are made to a system with all non-zero edges included which has been labeled the exact system. (A) Average difference in position between the exact solution and the approximation methods. (B) Average difference in velocity between the exact solution and the approximation solutions. (C) Potential energy, kinetic energy, and total energy of the system for the different methods. Note that there is about a half of unit difference between the thresholded (and combined) total energy and the exact (and sparsified) total energy. (D) Normalized second invariant of the stress tensor.

The surface relaxation problem (without the hole) is performed for 20,000 times steps which is approximately 4.5 periods based on (20). Fig 4 shows the energy of the system, the second invariant of the stress tensor, and the average error in position and velocity for this relaxation simulation. Results are compared to a system where neither spectral sparsification nor thresholding was applied which is referred to as the exact solution. These results illustrate that spectral sparsification has lower error than thresholding in position and velocity errors. As expected, sparsification better preserves the total energy of the system as seen in Fig 4(c). Also note that the difference of the stress invariant is negligible for the simulation.

**Fig 5:**
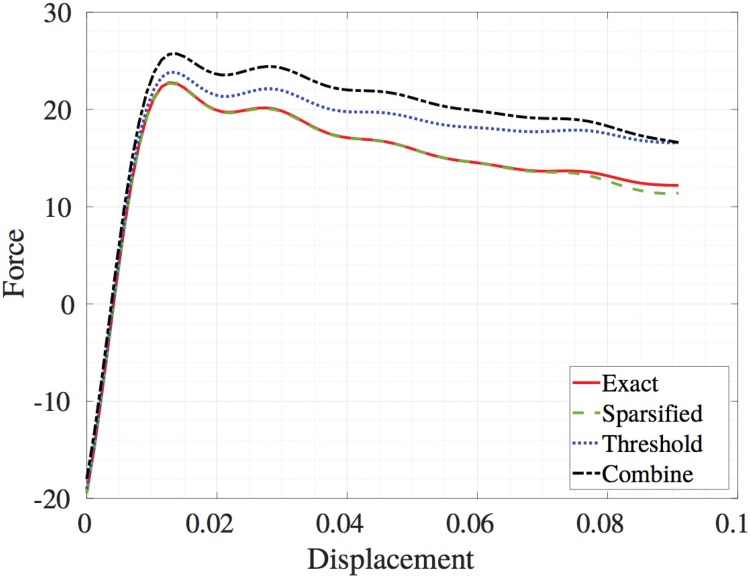
Force-displacement curve of the tensile simulation. Sparsification closely matches the force-displacement curve of the full graph (i.e., exact solution).

**Fig 6:**
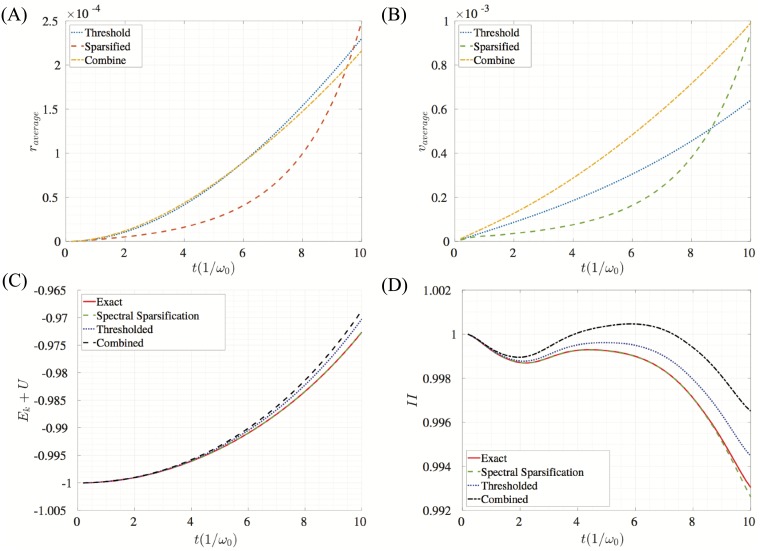
Measures of error and global properties for the sparsified, thresholded, and combined approximations for the tensile simulations. Errors are calculated by comparing sparsified networks to a network with no edge reduction. The network with no sparsification is labeled as exact. (A) Average difference in position between the exact solution and the approximation solutions. (B) Average difference in velocity between the exact solution and the approximation methods. (C) Potential energy, kinetic energy, and total energy of the system for the different methods. Note that there is about a half of unit difference between the thresholded (and combined) total energy and the exact (and sparsified) total energy. (D) Normalized second invariant of the stress tensor.

For the second validation case, we evaluate the tensile test of a solid with a circular hole on the side and invoke half symmetry boundary conditions of a hole inside a plate. The atoms on the top of the crystal are slowly pulled by controlling their displacement in the *y*-direction. The atoms on the left side and the bottom have the *x* and *y* positions held fixed, respectively, to ensure half symmetry. The displacement and force of the atoms during the tensile test are recorded and plotted in Fig 5. The fact that the sparsified system closely matches the force-displacement curve suggests that a sparsified system will better predict global properties such as elastic modulus and yield stress, which can be derived from the slope of the curve and its change in slope, respectively. As in the first example, we plot the average error in position and velocity as well as the global energy and stress invariant for the tensile test in Fig 6. We note that sparsification performs better in terms of energy of the system. Lower error in both position and velocity is achieved for up to 8 periods based on (20). We also note that the combined algorithm closely matches the thresholded algorithm. This is because the thresholding value decides the level of error in the combined algorithm.

## Conclusions

We have shown that spectral sparsification can provide a sparse representation of a material network by approximating the interatomic forces. Spectral sparsification produces a lower level of error relative to thresholding for problems containing long-range Coulomb forces. Importantly, the results suggest that sparsification maintains the net force. This is significant because it provides advantages for estimating macroscopic properties relative to thresholding. We tested this hypothesis on a simple tensile test and found the force-displacement curves for sparsification more closely resembled those of the full graph solution than thresholding for a model problem with a circular hole defect. We also presented a modification to the spectral sparsification algorithm that is fully parallelizable via spatial decomposition of the domain. Error propagation over several molecular dynamic time steps for a fixed spectral sparsification was shown to provide good estimates of many global characteristics such as total energy, average stress, and average atomic displacements for condensed atomic structures with relatively small perturbations of atomic positions. Molecular systems such as biological materials in water may undergo large configurational changes in shape. In such scenarios, the frequency of sparsification updates must be evaluated to assess accuracy. This will become important in highly nonlinear regimes and interactions containing more than two-body effects.

## Supporting information

S1 File **Section A**: Computational costs associated with spectral sparsification. Section B. Derivation of the angular frequency for a Lennard Jones potential. Fig A. (A) Scaling of resistance calculation where *t*_*r*_ is the time to perform the calculation of graph resistance. (B) Scaling of sampling calculation where *t*_*s*_ is the time to sample the edges. (C) Scaling simulation times, *t*_*d*_, vs. number of atoms using spectral sparsification for 100 time steps (D) Scaling simulation times, *t*_*d*_, vs. number of edges using spectral sparsification for 100 time steps. Fig B. Plots of the adjacency matrices created by (A) thresholding and (B) spectral sparsification. These two graphs have comparable error, but the spectrally sparsified system has 136,245 edges while the thresholded system has 307,532 edges and ignores the long range interactions of the Coulomb potential. Fig C. (A) Graphs of varying sparsity created by thresholding. The cutoff distance is given by the fraction of the maximum distance, *d*, across the domain consisting of ∼200 atoms. (B) Graphs of varying sparsity (*ϵ* = 0.25, 0.5 and 1) created by spectral sparsification again for ∼200 atoms. The sparsity of graphs which are in the same row position are equal.
(PDF)
