# Supplementary material for: Sparsification of long range force networks for molecular dynamics simulations
Source: PLoS One. 2019 Apr 12;14(4):e0213262. doi: 10.1371/journal.pone.0213262 (PMC6461233; doi:10.1371/journal.pone.0213262)
Supplement: S1 File — Section A: Computational costs associated with spectral sparsification. Section B. Derivation of the angular frequency for a Lennard Jones potential. Fig A. (A) Scaling of resistance calculation where tr is the time to perform the calculation of graph resistance. (B) Scaling of sampling calculation where ts is the time to sample the edges. (C) Scaling simulation times, td, vs. number of atoms using spectral sparsification for 100 time steps (D) Scaling simulation times, td, vs. number of edges using spectral sparsification for 100 time steps. Fig B. Plots of the adjacency matrices created by (A) thresholding and (B) spectral sparsification. These two graphs have comparable error, but the spectrally sparsified system has 136,245 edges while the thresholded system has 307,532 edges and ignores the long range interactions of the Coulomb potential. Fig C. (A) Graphs of varying sparsity created by thresholding. The cutoff distance is given by the fraction of the maximum distance, d, across the domain consisting of ∼200 atoms. (B) Graphs of varying sparsity (ϵ = 0.25, 0.5 and 1) created by spectral sparsification again for ∼200 atoms. The sparsity of graphs which are in the same row position are equal. (PDF) [file pone.0213262.s001.pdf]

Supplementary material: Sparsification of long range force networks for molecular dynamics simulations

Peter Woerner[1], Aditya G. Nair[1,2], Kunihiko Taira[1,3], William S. Oates[1*]

1 Department of Mechanical Engineering, Florida A&M-Florida State University College of Engineering, Tallahassee, FL, USA
2 Department of Mechanical Engineering, University of Washington, Seattle, WA, USA
3 Department of Mechanical and Aerospace Engineering, University of California, Los Angeles, CA, USA

*woates@fsu.edu

# Section A. Computational costs associated with spectral sparsification

We briefly describe the computational complexity associated with the spectral sparsification approach, which is comprised of the computation of effective resistance and a random sampling process. To compute the effective resistance, the Moore-Penrose pseudoinverse of the graph Laplacian needs to be determined which scales as $\mathcal{O}\left(n^2\right)$ as shown in Fig A. The random sampling procedure takes $\mathcal{O}\left(n^2 \log n\right)$ time. The computational time associated with molecular dynamics simulations after 100 time steps (iterations) as a function of the number of nodes and edges of the graph are shown in Fig A. As opposed to the original graph, which takes $\mathcal{O}\left(n^2\right)$ time equivalent to the number of edges in the complete graph, the computation time is reduced to $\mathcal{O}\left(n \log n\right)$ for the sparse graph, leading to computational savings.

Additional details on the reduction in complexity is illustrated by plotting the non-zero weights in the adjacency matrix as shown in Figs B and C for different levels of sparsification. In Fig B, a comparison of the reduction of edges is shown between thresholding and spectral sparsification. This figure highlights a signficant reduction of edges (almost 1/3 the amount of edges) for the spectrally sparsified graph while maintaining comparable errors for a Coulomb potential simulation. In Fig C, additional details on varying levels of sparsification are shown in terms of the adjacency matrix highlighting the difference in sparsity as a function of either the cutoff radius or the spectral sparsification level as controlled by $\epsilon$.

# Section B. Derivation of the angular frequency for a Lennard Jones potential

The angular frequency of a harmonic oscillator is given by

$$\omega_0 = \sqrt{\frac{k}{m}} \tag{1}$$

where $m$ is the mass of the particle and $k$ is the stiffness obtained from

$$k = \left.\frac{\partial^2 U}{\partial r^2}\right|_{r=r_0} \tag{2}$$

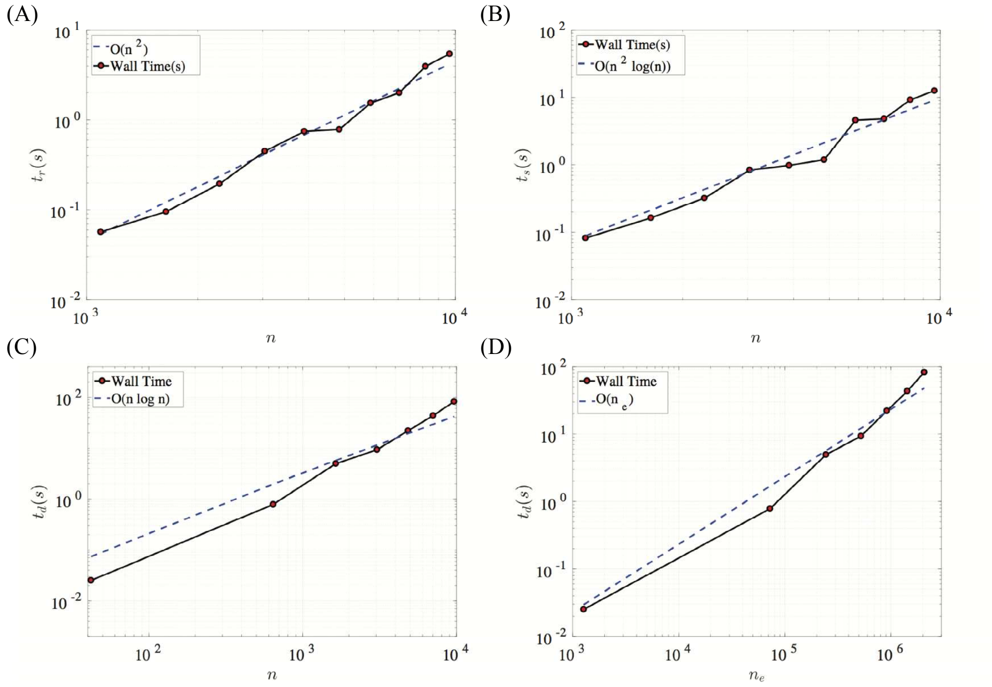


**Fig A.** (A) Scaling of resistance calculation where tr is the time to perform the calculation of graph resistance. (B) Scaling of sampling calculation where $t_s$ is the time to sample the edges. (C) Scaling simulation times, $t_d$, vs. number of atoms using spectral sparsification for 100 time steps (D) Scaling simulation times, $t_d$, vs. number of edges using spectral sparsification for 100 time steps.

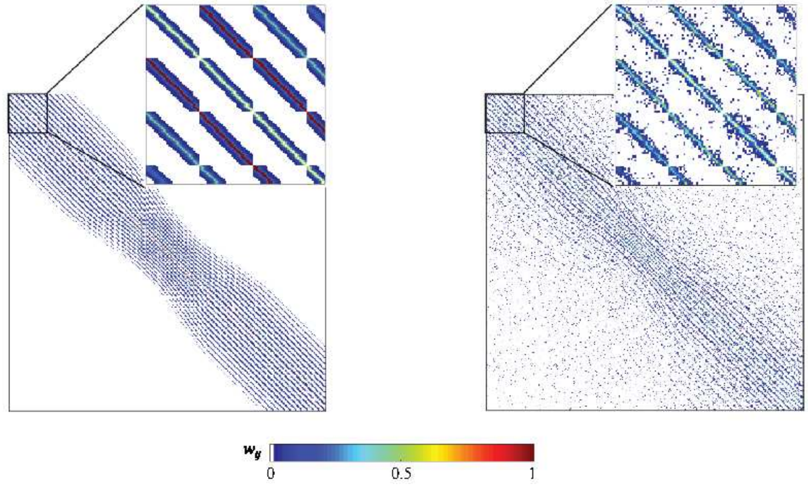


**Fig B.** Plots of the adjacency matrices created by (left) thresholding and (right) spectral sparsification. These two graphs have comparable error, but the spectrally sparsified system has 136,245 edges while the thresholded system has 307,532 edges and ignores the long range interactions of the Coulomb potential.

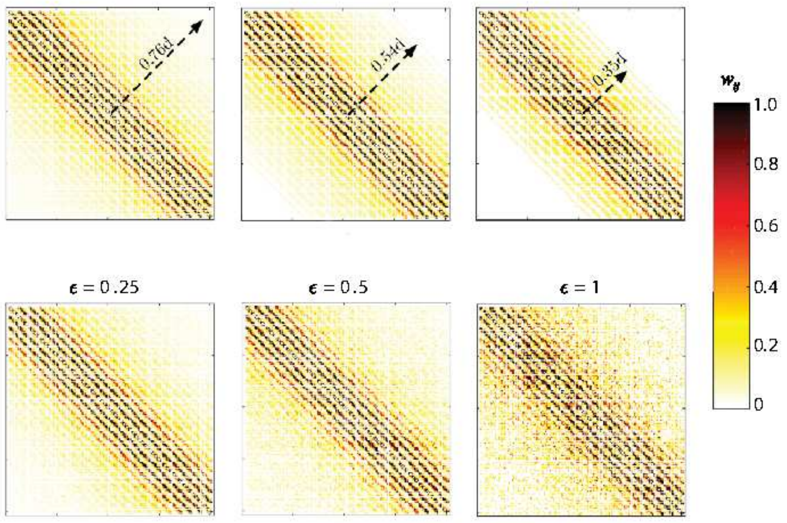


**Fig C.** (Top) Graphs of varying sparsity created by thresholding. The cutoff distance is given by the fraction of the maximum distance, $d$, across the domain consisting of ∼200 atoms. (Bottom) Graphs of varying sparsity ($\epsilon = 0.25, 0.5$ and 1) created by spectral sparsification again for ∼200 atoms. The sparsity of graphs which are in the same row position are equal.

where $r_0$ is the equilibrium position in the potential. In the case of the Lennard-Jones potential, we have

$$\begin{aligned} U &= 4\epsilon_0 \left[ \left(\frac{\sigma}{r}\right)^{12} - \left(\frac{\sigma}{r}\right)^{6} \right] \\ \frac{\partial U}{\partial r} &= \frac{48\epsilon_0}{\sigma} \left[ -\left(\frac{\sigma}{r}\right)^{13} + \frac{1}{2}\left(\frac{\sigma}{r}\right)^{7} \right] \\ \frac{\partial U^2}{\partial r^2} &= \frac{48\epsilon_0}{\sigma} \left[ 13\left(\frac{\sigma}{r}\right)^{14} - \frac{7}{2}\left(\frac{\sigma}{r}\right)^{8} \right] \end{aligned} \tag{3}$$

these yield

$$r_0 = 2^{\frac{1}{6}}\sigma \tag{4}$$

and

$$k = \frac{72\epsilon_0}{2^{1/3}\sigma^2}. \tag{5}$$
